# Characteristics of Drug‐Related Deaths Among Individuals Engaged in Sex Work in the United Kingdom, 1997–2023

**DOI:** 10.1111/dar.70008

**Published:** 2025-07-15

**Authors:** Emmert Roberts, Sharmila Parmanand, Caroline Copeland

**Affiliations:** ^1^ National Addiction Centre, Institute of Psychiatry, Psychology and Neuroscience King's College London London UK; ^2^ South London and the Maudsley NHS Foundation Trust London UK; ^3^ Department of Gender Studies London School of Economics London UK; ^4^ Faculty of Life Sciences & Medicine King's College London London UK

**Keywords:** benzodiazepines, drug‐related deaths, epidemiology, opioids, sex work

## Abstract

**Introduction:**

Individuals engaged in sex work are an understudied population recognised to be at differential risk of experiencing drug‐related harms. We aimed to determine the case characteristics, circumstances of death and type of implicated drugs among sex workers dying due to drug‐related causes.

**Methods:**

Retrospective cohort study in the United Kingdom using coronial records from the National Programme on Substance Use Mortality, 1997–2023. Information was available on decedent sociodemographics, characteristics of death and implicated drugs.

**Results:**

Nineteen decedents were reported to be sex workers at the time of their death. Overall, decedents were predominantly female (*n* = 17, 90%) with a mean age of 36.4 years (SD 8.0; range 26–58). Poisoning was the only disease or condition that was certified as a direct, antecedent or contributory cause of death. The mean number of drugs detected at post‐mortem was 5.4 (SD 2.5; range 1–10) with multiple drug toxicity implicated in the majority of cases (*n* = 18, 95%). The most commonly implicated drug groups were opioids (*n* = 17, 90%) and benzodiazepines (*n* = 9, 47%). All decedents had a history of substance dependence (*n* = 19, 100%), with almost a third injecting (*n* = 6, 32%).

**Discussion and Conclusions:**

There have been low but consistent numbers of drug‐related deaths each year where individuals were reported to be sex workers, results likely representing significantly conservative estimates. Polysubstance, opioid and benzodiazepine use are overrepresented within a largely female population with a significant burden of substance dependence. Non‐judgmental facilitation of access to evidence‐based addiction treatment, in particular for opioid use disorder, should be a priority.

**Summary**

- International research consistently reports an increased risk of drug‐related harm among individuals who are engaged in sex work.
- Over the last two decades in the UK, there have been 19 drug‐related deaths where individuals were reported to be sex workers.
- Overall decedents were predominantly female and all had a history of substance dependence. Opioids were implicated in death in the majority of cases.
- Non‐judgmental facilitation of access to evidence‐based addiction treatment, in particular for opioid use disorder, should be a priority.
- There are likely a substantial number of individuals engaged in sex work where this had either not been established by those submitting coronial evidence, or their sex work status was not deemed pertinent to report. As such, numbers reported are likely to represent significantly conservative estimates.

## 1 Introduction

In recent years, multiple countries have reported their highest number of drug‐related deaths since records began [1], with international research consistently reporting an increased risk of drug‐related harm among individuals who are engaged in sex work [2]. While ‘sex work’ can encompass a broad spectrum of individuals providing a range of sexual services, research persistently demonstrates that substantial barriers, including compounded stigma towards both drug use and sex work, can result in disproportionately reduced access to evidence‐based healthcare services among this population [3, 4, 5].

Despite an improved understanding of the drivers for increased prevalence of drug use among people engaged in sex work [6], and a recent greater emphasis on understanding healthcare inequities in potentially marginalised and vulnerable groups [7], there has been limited systematic epidemiological examination of drug‐related harms within this population. Few jurisdictions routinely collect or report drug‐related harms by occupational status and, when they do, they tend to do so using broad classes of occupation, and do not report on work conducted in informal economies [4]. Given the current epidemic of drug‐related deaths, if specific case characteristics and/or particular drug use profiles were found to be associated with fatalities among people engaged in sex work, this could lead to the development of targeted interventions or specific harm reduction advice.

To address these gaps, we aimed to conduct, to our knowledge, the first epidemiological study to determine: (i) the case characteristics; (ii) the circumstances of death; and (iii) the number and type of drugs implicated in death among people dying due to drug‐related causes who were reported to be sex workers at the time of their death in the United Kingdom.

## 2 Methods

This study is reported according to the Strengthening the Reporting of Observational Studies in Epidemiology (STROBE) statement [8], the completed checklist available as Table S1 in the Supporting Information.

### 2.1 The National Programme on Substance Use Mortality (NPSUM)

We conducted a retrospective cohort study using records drawn from the National Programme on Substance Use Mortality (NPSUM) [9]. This database contains information on an observational cohort that collects coronial data on deaths at any age related to any psychoactive drug, other than nicotine, caffeine or where the sole substance implicated is alcohol [10]. In total, NPSUM holds records on more than 60,000 deaths with reports received from England, Wales, the Channel Islands and the Isle of Man since 1997. Additional reports were received from the Scottish Crime and Drug Enforcement Agency between 2004 and 2011 and from the General Register Office for Northern Ireland since 2004. Deaths are referred to a coroner if the cause of death is unknown, is violent or unnatural, sudden and unexplained, occurred during or following a period of anaesthesia or may have been caused by an industrial disease or poisoning [11]. Coronial files typically include the coroner's decision as to cause of death, statements from witnesses, family and friends, first responders (e.g., police, emergency services), general practitioner records and hospital records. Toxicology is discretionally requested by individual coroners, and coronial jurisdictions choose to voluntarily report all deaths within their area to NPSUM if any of the following criteria are met: (i) psychoactive substance(s) are directly implicated in death; (ii) an individual has a history of dependence or misuse of drugs; or (iii) if controlled drugs are identified at post‐mortem. The King's College London Biomedical and Health Sciences, Dentistry, Medicine and Natural and Mathematical Sciences Research Ethics Subcommittee re‐confirmed in August 2024 that NPSUM does not require Research Ethics Committee review, as all individuals are deceased.

### 2.2 Case Identification

Due to the lagged nature of mortality data complete case records were available between 1997 and April 2023. All cases were identified and NPSUM fields searched for the following terms: ‘sex‐work*’, ‘sex work*’, hooker*, streetwalker*, whore*, strip*, ‘exotic dancer’, prostitute*, escort*, ‘call girl’*, brothel*, ‘rent boy*’, gigolo*, masseuse*, masseur*, dominatrix, sauna, only fans and madam. We note these terms were selected in collaboration with people with lived experience and are intended to capture all cases, their selection does not represent endorsement of their normative usage. Authors manually reviewed the text of coronial files to confirm each decedent was reported to be a sex worker at the time of their death. Final searches were conducted on 8 December 2024.

### 2.3 Measures

Data on decedent sociodemographics, mental health history (i.e., a documented history of individual mental health conditions), substance use history (i.e., documented history of substance dependence and injecting status) and which individual substances were ultimately deemed implicated in death were extracted from coronial files. All cases additionally recorded the findings concerning the disease or condition that led directly to death, any antecedent cause(s) and other significant conditions contributory to death.

### 2.4 Statistical Analysis

We generated descriptive statistics for all decedents. Means, standard deviations (SD) and ranges were presented for continuous variables, as well as median and interquartile range (IQR) in skewed distributions. Percentages were reported for binary and categorical variables. All analyses were conducted in STATA IC version 16. The analysis plan was not pre‐registered and the results should be considered exploratory.

## 3 Results

A total of 19 decedents were reported to be sex workers at the time of their death following drug use in the UK between 1997 and 2023.

### 3.1 Sociodemographics and Case Characteristics

Sociodemographic and case characteristics of all decedents can be found in Table 1. Overall, decedents were predominantly female (*n* = 17, 89.5%), of White ethnicity (*n* = 15, 78.9%), had a mean age of 36.4 years (SD 8.0; range 26–58) and a substantial number were reported as formally unemployed at the time of their death (*n* = 13, 68.4%). More than half of decedents died while in their own accommodation (*n* = 10, 52.6%). In only one case, in which death occurred outside of a hospital setting, was death reported to have occurred while another person was present, that person being reportedly identified as a client.

**TABLE 1:** Sociodemographic, case characteristics, circumstances of death and mental health and substance use history of people dying due to drug‐related causes reported to be engaged in sex work at the time of their death in the United Kingdom, 1997–2023.

	All, *n* = 19 *n* (%)			All, *n* = 19 *n* (%)	
Age	Mean, years	36.4 (SD 8.0; range 26–58)	Place of death	Own place of residence	10 (52.6)
Sex	Female	17 (89.5)		Other residential address	3 (15.8)
	Male	2 (10.5)		Hostel	2 (10.5)
Ethnicity	White	15 (78.9)		Hospital	3 (15.8)
	Black Caribbean	1 (5.3)		Not reported	1 (5.3)
	Asian	1 (5.3)	Direct cause of death (ICD‐10 code)	Poisoning, accidental (X40‐X45)	11 (57.9)
	Polynesian	1 (5.3)		Poisoning, intentional (X60‐X84)	1 (5.3)
	Unknown/not recorded	1 (5.3)		Poisoning, undetermined intent (Y10‐Y14)	2 (10.5)
Occupation^a^	Employed (Manual)	2 (10.5)		Unspecified multiple injuries (T07)	1 (5.3)
	Employed (Non‐manual)	1 (5.3)		Unascertained (R99) / Not reported	4 (21.1)
	Unemployed	13 (68.4)	Mental health	People with a history of any mental health disorder	10 (52.6)
	Student	1 (5.3)		People with a history of a depressive disorder	6 (31.6)
	Unknown	2 (10.5)		People with a history of an anxiety disorder	2 (10.5)
Year of death	1999–2003	6 (31.6)	Addiction	People with a history of substance dependence	19 (100.0)
	2004–2008	2 (10.5)		People with an injecting history	6 (31.6)
	2009–2013	5 (26.3)			
	2014–2018	3 (15.8)			
	2019–2023	4 (21.1)			

Abbreviations: ICD‐10, International Classification of Diseases, Tenth Revision; SD, standard deviation.

^a^ Occupation definitions are based on those used by the United Kingdom Office of National Statistics [12].

### 3.2 Characteristics of Death and Mental Health and Substance Use History

Circumstances of death and mental health and substance use histories of all decedents can be found in Table 1. Poisoning was the only disease or condition that was certified as a direct or antecedent cause or a contributory factor to death, with coronial determination reporting 11 deaths directly due to accidental poisoning (57.9%, International Classification of Diseases, Tenth Revision [ICD‐10] codes, X41, X42 and X45), one death due to intentional self‐poisoning (5.4%; ICD‐10, X61) and the remainder deemed of undetermined intent (36.8%; ICD‐10, Y12, R99). One death was concluded as an accident directly due to multiple injuries sustained following a fall (ICD‐10, T07).

In cases where medical history was available, over half of all decedents had a history of any mental health condition (*n* = 10, 52.6%), the two most common documented mental health conditions being a depressive disorder or an anxiety disorder. All decedents had a history of substance dependence (*n* = 19, 100.0%), with almost a third of decedents having a history of injecting (*n* = 6, 31.6%).

### 3.3 Number and Type of Drugs Implicated in Death

The number and type of drugs implicated in death can be found in Table 2. The median number of drugs detected at post‐mortem was 6 (interquartile range 3, 7) with multiple drug toxicity implicated in all but one case (*n* = 18, 94.7%). The two most common drug groups implicated in death were opioids (*n* = 17, 89.5%) and benzodiazepines (*n* = 9, 47.4%) with the three most common individual drugs implicated being cocaine (*n* = 8, 42.1%), heroin (*n* = 7, 36.8%) and diazepam (*n* = 7, 36.8%). A minority of decedents had a drug for which they had a prescription implicated in death (*n* = 4, 21.1%).

**TABLE 2:** The number and type of drugs implicated in death among people dying due to drug‐related causes reported to be engaged in sex work at the time of their death in the United Kingdom, 19,971–2023.

Drug implicated in death^a^		All, *n* = 19 *n* (%)
All	Mean number of drugs implicated	5.4 (SD 2.5; range 1–10)
	Median number of drugs implicated	6 (IQR 3, 7)
	Only single substance implicated	1 (5.3)
	Multiple substances implicated	18 (94.7)
Prescribed	Any prescribed drug implicated	4 (21.1)
Opioids	Any opioid	17 (89.5)
	Heroin	7 (36.8)
	Methadone	6 (31.6)
Benzodiazepines	Any benzodiazepine	9 (47.4)
	Diazepam	7 (36.8)
Antidepressants	Any antidepressant	4 (21.1)
Cocaine	Cocaine	8 (42.1)
Alcohol	Alcohol^b^	4 (21.1)

Abbreviations: IQR, interquartile range; SD, standard deviation.

^a^ Only those substances implicated in four or more deaths are reported.

^b^ The nature of the National Programme on Substance Use Mortality (NPSUM) database is such that deaths in which alcohol is the only substance implicated are not recorded. As such, drug‐related deaths in which alcohol is implicated in NPSUM have, by definition, multiple substances implicated.

## 4 Discussion

Over the last two decades within the NPSUM cohort, there has been low but consistent numbers of deaths following drug use, where individuals were reported to be sex workers at the time of their death. While the case characteristics are, in some ways, typical of UK drug‐related deaths over the same timeframe, including a high proportion of ethnically White decedents reported as being formally unemployed, atypically almost all decedents were female, all had a history of substance dependence, and there were no decedents with any physical or mental conditions or co‐morbidities deemed contributory to death [13].

Opioids and multiple drug toxicity were implicated in almost all fatalities, with a documented history of substance dependence reported in all cases. The preponderance of polysubstance, opioid, benzodiazepine, and cocaine use is similar to drug‐use profiles seen in other Western samples of individuals engaged in sex work [2]; however, the overwhelming proportion of deaths implicating opioid use, alongside substance dependence and an injecting history, is particularly striking. Previous research has consistently demonstrated that individuals who use drugs and are engaged in sex work face significant and unique barriers to access evidence‐based addiction treatment, with compounded stigma, lack of access to essential information, and fear of criminal penalties among documented reasons [5, 14]. These results provide advocacy for the identification of these individuals through non‐judgmental routine discussion of sex work in addiction treatment settings, and non‐judgmental routine discussion of potential drug use in non‐addiction settings in which people engaged in sex work may present [5]. Identification, particularly of those injecting or using opioids, and rapid facilitation of access to evidence‐based harm reduction and addiction treatment, in particular opiate agonist therapy [15], may significantly reduce the mortality risk in this ostensibly physically healthy population. Previous work has consistently highlighted the utility of including people with lived and living experience of both sex work and drug use in treatment policy and service design, with improved levels of trust, access and outcomes demonstrated for these groups when meaningful involvement has been facilitated [3, 14].

Contextually, it is also notable that in only one case did the fatal drug use occur while the sex worker was in the presence of another person, in this case reportedly identified as a client. This finding perhaps highlights the need to reinforce that professionals ensure this group is not being excluded from harm‐reduction messaging that advises people who use drugs to try and minimise the number of occasions on which the use alone [9, 15].

The study has several strengths, including the granular examination of substances implicated in death and the large time frame. There are, however, several important limitations. In order to identify individuals as engaged in sex work, the study relies on documentation submitted to the coroner to both contain this information and for it to be accurate [4]. There are likely a substantial number of individuals within NPSUM who were engaged in sex work where this had either not been established by those submitting coronial evidence or their sex work status was not deemed pertinent to report. Within the UK, the exchange of sexual services for money is legal in all devolved nations, except for Northern Ireland; however, some activities related to sex work (e.g., solicitation in public areas) remain criminal offences, potentially impacting reporting practices [16]. Similarly, many individuals may have been historically engaged in sex work but not at the point of their death. As such, the numbers reported in this study are likely to represent significantly conservative estimates. Nevertheless, to our knowledge, this is the largest epidemiological study to examine drug‐related deaths within this population, and the sociodemographic, case characteristics, and implicated drug profiles have material utility to provide information on this often marginalised and under‐researched group. A further limitation within NPSUM is that potential variables of interest may either not be available or their quality and completion inconsistent across coronial jurisdictions. There are significant proportions of incomplete reporting for some variables (e.g., over a third of cases contain no reported occupation), which limits the ability to comment on key factors that may expose significant inequities. NPSUM also does not contain information on which decedents were accessing evidence‐based harm reduction or addiction treatment at their time of death; given the overwhelming proportion of decedents with a history of substance dependence, knowledge of the proportion of people accessing these services would be valuable. Future data linkage to treatment records may thus be a fruitful avenue for further study. Additionally, we did not consult coronial staff on the search terms used, and due to the voluntary submission nature, NPSUM has incomplete coverage of the UK coronial system over the studied time frame, with variability of toxicological examination in all cases; thus, some drug‐related deaths will likely have been missed, and some implicated substances not identified [9].

In summary, there have been consistent numbers of drug‐related deaths where decedents were reported to be sex workers over the past two decades. Polysubstance, opioid, benzodiazepine, and cocaine use are overrepresented within a largely female population suffering from minimal physical health problems, but with a significant burden of substance dependence and injecting. Meaningful inclusion in treatment policy and service design, and the rapid facilitation of access to evidence‐based addiction treatment, and in particular treatment for opioid use disorder, should be a priority for this group given the significant impact on mortality risk reduction and the substantial barriers to treatment often faced by these marginalised individuals [3, 15].

## Author Contributions

Each author certifies that their contribution to this work meets the standards of the International Committee of Medical Journal Editors.

## Conflicts of Interest

The authors declare no conflicts of interest.

## Supporting information


**Data S1** Supporting Information.

## Data Availability

The data that support the findings of this study are available on request from the corresponding author. The data are not publicly available due to privacy or ethical restrictions.
