# Supplementary material for: Characteristics of Drug‐Related Deaths Among Individuals Engaged in Sex Work in the United Kingdom, 1997–2023
Source: Drug Alcohol Rev. 2025 Jul 15;44(6):1805–10. doi: 10.1111/dar.70008 (PMC12405828; doi:10.1111/dar.70008)
Supplement: Supplementary file 1 — Data S1 Supporting Information. [file DAR-44-1805-s001.docx]

Table S1. Strengthening the Reporting of Observational Studies in Epidemiology (STROBE) statement checklist [6]

|  | Item No | Recommendation |
| --- | --- | --- |
| **Title and abstract** | 1 | (*a*) Indicate the study’s design with a commonly used term in the title or the abstract; Title: Page 1 |
|  |  | (*b*) Provide in the abstract an informative and balanced summary of what was done and what was found; Abstract: Page 3 |
| Introduction | | |
| Background/rationale | 2 | Explain the scientific background and rationale for the investigation being reported; Introduction: Page 5 |
| Objectives | 3 | State specific objectives, including any prespecified hypotheses; Introduction: Page 5 |
| Methods | | |
| Study design | 4 | Present key elements of study design early in the paper; Methods: Page 6/7 |
| Setting | 5 | Describe the setting, locations, and relevant dates, including periods of recruitment, exposure, follow-up, and data collection; Methods: Pages 6/7 |
| Participants | 6 | (*a*) Give the eligibility criteria, and the sources and methods of selection of participants. Describe methods of follow-up; Methods: Pages 6/7 |
|  |  | (*b*) For matched studies, give matching criteria and number of exposed and unexposed N/A |
| Variables | 7 | Clearly define all outcomes, exposures, predictors, potential confounders, and effect modifiers. Give diagnostic criteria, if applicable Methods: Page 6/7 |
| Data sources/ measurement | 8* | For each variable of interest, give sources of data and details of methods of assessment (measurement). Describe comparability of assessment methods if there is more than one group Methods: Page 6/7 |
| Bias | 9 | Describe any efforts to address potential sources of bias Methods: Page 6/7 |
| Study size | 10 | Explain how the study size was arrived at Methods Page 6/7 ; Results Page 9 |
| Quantitative variables | 11 | Explain how quantitative variables were handled in the analyses. If applicable, describe which groupings were chosen and why Methods Page 7 |
| Statistical methods | 12 | (*a*) Describe all statistical methods, including those used to control for confounding Methods Page 7 |
|  |  | (*b*) Describe any methods used to examine subgroups and interactions N/A |
|  |  | (*c*) Explain how missing data were addressed Methods Page 6/7 |
|  |  | (*d*) If applicable, explain how loss to follow-up was addressed N/A |
|  |  | (*e*) Describe any sensitivity analyses N/A |
| Results | | |
| Participants | 13* | (a) Report numbers of individuals at each stage of study—eg numbers potentially eligible, examined for eligibility, confirmed eligible, included in the study, completing follow-up, and analysed Results Page 9 |
|  |  | (b) Give reasons for non-participation at each stage N/A |
|  |  | (c) Consider use of a flow diagram N/A |
| Descriptive data | 14* | (a) Give characteristics of study participants (eg demographic, clinical, social) and information on exposures and potential confounders Results Pages 9/10 (Table 1) |
|  |  | (b) Indicate number of participants with missing data for each variable of interest Table 1 |
|  |  | (c) Summarise follow-up time (eg, average and total amount) Methods Page 7/8 |
| Outcome data | 15* | Report numbers of outcome events or summary measures over time Table 1 |
| Main results | 16 | (*a*) Give unadjusted estimates and, if applicable, confounder-adjusted estimates and their precision (eg, 95% confidence interval). Make clear which confounders were adjusted for and why they were included Table 1 |
|  |  | (*b*) Report category boundaries when continuous variables were categorized Table 1 |
|  |  | (*c*) If relevant, consider translating estimates of relative risk into absolute risk for a meaningful time period N/A |
| Other analyses | 17 | Report other analyses done—eg analyses of subgroups and interactions, and sensitivity analyses Table 1 Results Pages 9/10 |
| Discussion | | |
| Key results | 18 | Summarise key results with reference to study objectives Discussion Page 11 |
| Limitations | 19 | Discuss limitations of the study, taking into account sources of potential bias or imprecision. Discuss both direction and magnitude of any potential bias Discussion Page 11/12 |
| Interpretation | 20 | Give a cautious overall interpretation of results considering objectives, limitations, multiplicity of analyses, results from similar studies, and other relevant evidence Discussion Page 11 |
| Generalisability | 21 | Discuss the generalisability (external validity) of the study results Discussion Page 11/12 |
| Other information | | |
| Funding | 22 | Give the source of funding and the role of the funders for the present study and, if applicable, for the original study on which the present article is based Title Page 2 |
